# Association between Metabolic Syndrome and Osteoporosis: A Systematic Review and Meta-Analysis

**DOI:** 10.1155/2021/6691487

**Published:** 2021-07-16

**Authors:** Weida Liu, Chuangshi Wang, Jun Hao, Lu Yin, Yang Wang, Wei Li

**Affiliations:** Medical Research & Biometrics Center, Fuwai Hospital, State Key Laboratory of Cardiovascular Disease, National Center for Cardiovascular Diseases, Peking Union Medical College and Chinese Academy of Medical Sciences, Beijing 102300, China

## Abstract

**Background:**

Previous studies have reached mixed conclusions regarding the association between metabolic syndrome (MS) and osteoporosis. We aimed to perform a meta-analysis based on published studies that explored the association between osteoporosis and MS.

**Methods:**

To identify related literature, a systematic search of PubMed, Cochrane Library, and EMBASE databases from inception to June 2020 was performed. Original studies that reported the risk estimates of osteoporosis morbidity for two or three categories of bone mineral density (BMD) in patients with MS were selected. Two independent investigators screened and selected the articles. Summary odds ratios (ORs) and 95% confidence intervals (CIs) were calculated using random-effects models.

**Results:**

Of 2632 identified studies, nine cross-sectional studies with 14 datasets were eligible for our meta-analysis. In seven studies (10 datasets), the summarized ORs of osteoporosis for MS were 0.72 (95% CI: 0.52–0.99). Subgroup analyses by gender showed that significant inverse associations were observed only in men (OR = 0.72, 95% CI: 0.55–0.96) but not in women (OR = 0.70, 95% CI: 0.41–1.22). The definition of MS, the source of the study population, and the adjustment of covariates affected the estimates. In two studies (4 datasets), there was no evidence for an association between MS and decreased BMD.

**Conclusions:**

Our findings demonstrated that MS was significantly associated with a lower osteoporosis risk. There might be gender differences in the association between MS and osteoporosis. In addition, the association was likely to relate to the definition of MS, the source of the study population, and the adjustment of covariates.

## 1. Background

Metabolic syndrome (MS) is a cluster of conditions characterized by abdominal obesity, hypertension, insulin resistance, and dyslipidemia (elevated triglyceride (TG) and low high-density lipoprotein cholesterol (HDL-C) levels) [1]. From the perspectives of both individual clinicians and the health of the general public, MS is an attributable risk to the epidemic of diabetes, stroke, and coronary heart disease [2–4]. With increasing longevity, the number of people who are at risk for MS is progressively increasing in most countries. In the US National Health and Nutrition Examination Survey, more than one in three adults have MS [5].

Osteoporosis, regarded as reduced bone mineral density (BMD) and quality and an increased risk of fractures, is one of the most common chronic diseases, affecting nearly 200 million people worldwide [6]. In order to define osteoporosis, the World Health Organization (WHO) has categorized potential patients into 3 classifications according to changes in BMD obtained by dual-energy X-ray absorptiometry (DEXA) *T* score: normal BMD (+1 to −1), osteopenia or low bone mass (−1 to −2.5), and osteoporosis (−2.5 or below) [7].

As the world population is progressively aging, MS and osteoporosis are more likely to coexist in the same patient [8], which may have an impact both on the quality of life and on healthcare resources. Studies have shown that insulin resistance and bone metabolism are linked. Insulin signaling regulates osteoclast differentiation and osteoblast activity [9, 10], which may damage the bone quality and cause the development of osteoporosis. Two relevant meta-analyses on the association between MS and BMD were published previously [11, 12]. However, according to the WHO diagnostic criteria, we consider it is more clinically significant that BMD (continuity variable) is divided into osteoporosis and osteopenia (categorical variable). In addition, three studies on the association of MS and osteoporosis were published recently [13–15].

Therefore, we performed a systematic review and meta-analysis of available studies that assess the association between MS and osteoporosis in the general population. Our objective was to explore whether there is an association between MS and osteoporosis and to find out whether there are any factors that may affect these associations.

## 2. Methods

### 2.1. Search Strategy

PubMed, Cochrane Library, and EMBASE databases were searched from inception to June 2020 using the following terms: “metabolic syndrome” or “insulin resistance syndrome” or “plurimetabolic syndrome” or “syndrome X” or “MS” and “osteoporosis” or “osteopenia” or “bone mineral density” or “BMD” or “bone density” or “bone mineral contents” or “metabolic bone diseases.” Reference lists of retrieved articles and relevant reviews were manually searched. In September 2020, the databases were searched again using the same search criteria for additional studies. No language restrictions and study design restrictions were applied.

### 2.2. Eligibility and Study Selection

Inclusion criteria for our study were as follows: studies that were published as original reports, studies that reported the diagnostic criteria of MS, studies that reported the diagnostic criteria of osteoporosis, studies that reported the outcome of interest as osteoporosis or osteopenia (excluded studies that reported only BMD), and studies that reported risk estimates of osteoporosis or osteopenia and their corresponding 95% confidence interval (CI) for MS (or data to calculate them).

Title and abstract screening were performed for each article to remove obviously irrelevant and duplicated reports. Articles deemed potentially eligible by title and abstract screening were reexamined by full-text review according to the above inclusion criteria. The eligibility of articles was finally determined by 2 independent authors. Any discrepancies were resolved through discussion. For studies appearing in more than one publication, the most recent publication was included to avoid duplicate observation, unless more inclusive or detailed data were found in other publications.

### 2.3. Data Extraction

Data were extracted using a standardized data collection form. The following items were extracted from each study: first author's surname, publication year, country or region of the study origin, study design, number of participants, gender, mean age, the detection method of BMD, site of BMD, diagnostic criteria of MS, diagnostic criteria of osteoporosis or osteopenia, risk estimates and their corresponding 95% CI, and adjustment for potentially confounding factors. The models with the most covariate adjustment from each study were selected and used for the meta-analysis. If a study did not clearly mention any above key points, we contacted the authors of the primary reports to request any unpublished data (we contacted six authors for clarification and to obtain further data and one replied). Discrepancies were resolved by discussion.

### 2.4. Assessment of Bias Risk

A subjective assessment of methodological quality for observational studies was evaluated by two authors using the Agency for Health Care Research and Quality (AHRQ) method list, which is a quality assessment tool for the cross-sectional study [16]. Eleven questions were answered. If the answer is “No” or “Unclear,” the score of the item is “0;” if the answer is “Yes,” the score of the item is “1.” The quality of the study is evaluated as follows: low quality = 0 − 3; moderate quality = 4 − 7; high quality = 8 − 11.

### 2.5. Statistical Analysis

Study-specific findings were combined using the random-effects model by DerSimonian and Laird. We estimated unadjusted risk estimates using the reported numbers of participants for the studies in which only this information and no model results were published. Heterogeneity across studies was examined by using the *Q* and *I*^*2*^ statistic (significance level at *p* < 0.10) [17]. Because clinical characteristics were not consistent between men and women, all our analyses are compared by gender. A sensitivity analysis was performed to assess the influence of the individual studies on the overall results by omitting one study at a time. Subgroup analysis was performed to find factors that may explain heterogeneity or difference in outcome between each study. Potential publication bias was assessed by Egger's test and Begg's test. All analyses were performed using STATA version 12.0.

## 3. Results

### 3.1. Literature Search and Study Characteristics

**Figure 1:**
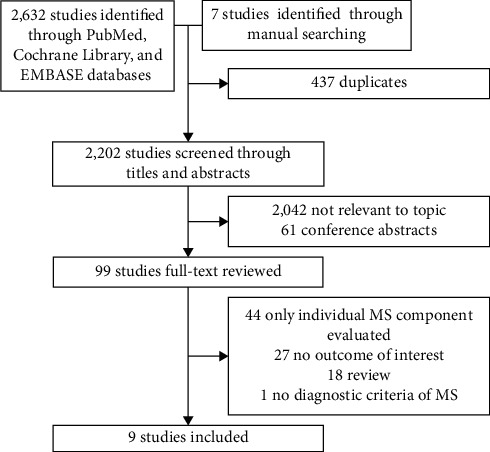
Results of information search.

A total of 2,632 references were identified following an electronic search and seven were identified by manual searching, of which 437 were duplicates, 2,042 were not relevant, and 61 were conference abstracts and so were excluded at the initial screening of the title and abstract. By full-text review, 90 more studies were excluded: 44 studies only reported the component of MS, 27 studies did not report the outcome of interest, 18 studies were reviews, and one study did not report the diagnostic criteria of MS. The complete study selection process is described in Figure 1.

Ultimately, nine cross-sectional studies with 12,987 individuals were eligible for our meta-analysis [13–15, 18–23]. Seven studies compared the association between MS and osteoporosis, and two studies compared the association between MS and decreased BMD (included both osteopenia and osteoporosis). A summary of the included studies is given in Table 1. Included studies were published between 2013 and 2018 and were performed in China, Iran, Germany, Korea, and Morocco. The mean age ranged from 52 to 71 years. Five studies reported multiple datasets by gender, one study only reported male participants, and the remaining three only reported female participants. MS was diagnosed according to the National Cholesterol Education Program's Adult Treatment Panel III (NCEPATP III) [24] in six studies, the Chinese Diabetes Society (CDS) [25] in one study, and harmonization criteria [26] in two studies. All studies measured BMD by DEXA and diagnosed osteoporosis according to the WHO definition.

As given in Table 2, the adjusted confounders varied across the included studies. Six studies adjusted for a wide range of risk factors for osteoporosis, including age, body mass index (BMI), calcium intake, physical activity, educational level, alkaline phosphatase, nonalcoholic fatty liver disease, smoking status, alcohol consumption, and so on.

### 3.2. Quality Assessment of Included Studies

The quality of the included studies was evaluated by the AHRQ list (Table 3). The total score ranged from 1 to 11. All studies had high or moderate quality (mean score = 8.1).

### 3.3. The Association between MS and Osteoporosis

**Figure 2:**
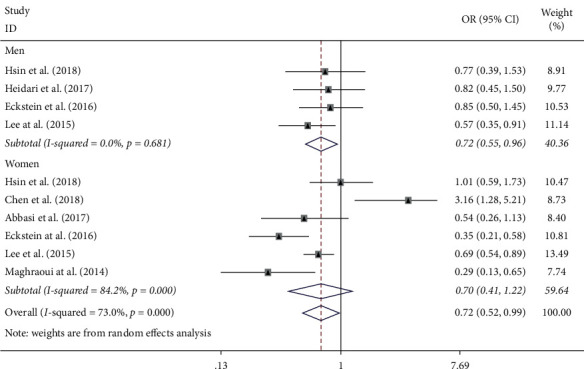
Forest plot showing the association between MS and the risk of osteoporosis (osteoporosis vs. nonosteoporosis).

As shown in Figure 2, ten datasets compared nonosteoporosis to osteoporosis with or without MS; there was a borderline significant association between MS and osteoporosis (OR = 0.72, 95% CI: 0.52–0.99, *p*=0.046) when combining all ORs with the random-effects model, and a high degree of heterogeneity (*I*^*2*^ = 73%, *p* < 0.001) between these studies. The result of Egger's test (*p*=0.198) and Begg's test (*p*=0.869) does not indicate publication bias among studies of MS and osteoporosis. Sensitivity analysis (one study was omitted per round) did not significantly alter the results, ranging from 0.63 (95% CI: 0.50–0.80) to 0.78 (95% CI: 0.57–1.08).

Notably, subgroup analyses by gender revealed that a significant inverse relationship was observed merely in men (OR = 0.72, 95% CI: 0.55–0.96, *p*=0.023) but not in women (OR = 0.70, 95% CI: 0.41–1.22, *p*=0.210). There was no evidence of statistical heterogeneity in the male subgroup (*I*^*2*^ = 0%, *p*=0.681) but substantial heterogeneity in the female subgroup (*I*^*2*^ = 84.2%, *p* < 0.001).

### 3.4. The Association between MS and Decreased BMD

**Figure 3:**
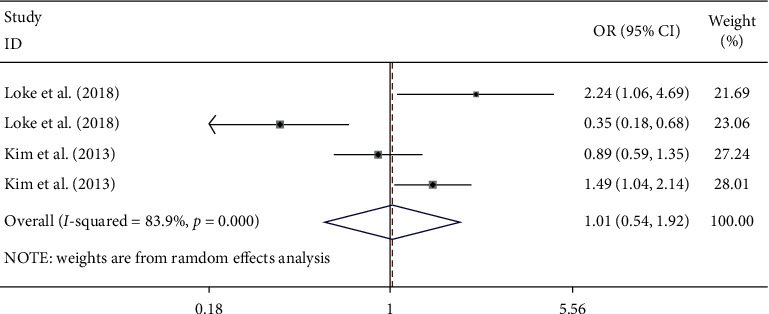
Forest plot showing the association between MS and the risk of decreased BMD.

As shown in Figure 3, four datasets compared normal BMD to decreased BMD (includes both osteopenia and osteoporosis) with or without MS; there was no evidence for an association between MS and decreased BMD (OR = 1.01, 95% CI: 0.54–1.92, *p*=0.969) when combining all ORs with the random-effects model. High heterogeneity (*I*^*2*^ = 83.9%, *p* < 0.001) existed among the studies.

### 3.5. Subgroup Analyses

As given in Table 4, heterogeneity was further explored by subgroup analyses based on the definition of MS (NCEPATP III vs. CDS vs. harmonization criteria), country (Asian vs. non-Asian countries), studies' quality (high vs. moderate), and confounding factors (adjusted vs. unadjusted).

There was a significant association between MS and osteoporosis in MS defined by NCEPATP III (OR = 0.67, 95% CI: 0.54–0.84, *p* < 0.001) in a non-Asian country (OR = 0.46, 95% CI: 0.23–0.89, *p*=0.021) and in an unadjusted model (OR = 0.54, 95% CI: 0.31–0.95, *p* < 0.033).

## 4. Discussion

This systematic review and meta-analysis found that MS was associated with reduced osteoporosis risk in men but not in women. In addition, the association between osteoporosis and MS was likely to relate to the definition of MS, the source of the study population, and the adjustment of covariates.

Two relevant meta-analyses on the association between MS and BMD were published previously [11, 12]. The meta-analysis from Xue et al. showed that MS may have a beneficial influence on BMD, and the meta-analysis by Zhou et al. showed a negative effect of MS on BMD in men but not in women. However, according to the WHO diagnostic criteria, we consider it is more clinically significant that BMD (continuity variable) is divided into osteoporosis and osteopenia (categorical variable). Osteopenia is a transition from normal BMD to osteoporosis, which is generally considered a reversible process [27]. Our meta-analysis compared normal BMD to decreased BMD (includes both osteopenia and osteoporosis), and there was no evidence for an association between MS and decreased BMD; however, the result was not robust because of the small number of studies and high heterogeneity.

Our analysis compared nonosteoporosis to osteoporosis and suggested a positive effect of MS on osteoporosis. Two relevant meta-analyses from Esposito et al. [28] and Yang et al. [29] also reported that MS was significantly associated with a lower fracture risk. MS is a cluster of diseases consisting of several disorders; the mechanism following the impacts of MS on osteoporosis is intricate and has not yet been studied thoroughly. The relationship between the various components of MS and osteoporosis has been widely investigated, but the results are inconclusive. As the component of MS, the results of studies on the association between obesity and osteoporosis are mostly inconsistent. Some studies reported obesity was a protective effect for osteoporosis in several studies generally [30, 31]. Because it is associated with a higher 17*β*-estradiol level and higher mechanical load, which may improve bone density. Other studies [32–36] have pointed out that inflammation-related factors released by visceral adipose tissue increase the risk of osteoporosis by inhibiting bone formation. Abnormality of calcium metabolism is a key factor linking hypertension and osteoporosis. Zhang et al. [37] found hypertension was independently and significantly associated with osteoporosis, while Mussolino et al. [38] found no significant association between blood pressure and BMD at any bone site. Additionally, there are also controversial reports on the effects of dyslipidemia [39–41] and insulin resistance [32, 42] on bone health. We believe that both positive and negative influences of the MS components on the bone exist in parallel. Therefore, the combined effect of the MS components on osteoporosis could be positive or insignificant.

The gender-specific differences regarding the association between MS and osteoporosis were observed in our analysis. Menopause may be the reason why there was no significant association between MS and osteoporosis for women. The majority of women included in our meta-analysis were older and postmenopausal. Menopause is one of the major risk factors for osteoporosis partly due to reduced estrogen production [43], which may dilute the benefit of MS on the bone. In addition, several study-level variables leading to heterogeneity were further found by subgroup analyses, such as the MS definition, the source of the study population, the quality of the study, and the adjustment of covariates. First, we obtained a significant association between MS and osteoporosis in the subjects diagnosed by NCEPATP III criteria rather than other criteria (includes CDS criteria and harmonization criteria). According to NCEPATP III or other criteria, participants were diagnosed with MS when they had three or more of the following: obesity, hypertension, low HDL-C levels, high fasting blood glucose, or high triglycerides. Although the other two criteria were similar to the NCEPATP III criteria, there were still differences. For example, the NCEPATP III criteria defined obesity by waist circumference, while the CDS defined obesity by BMI. Second, we obtained a significant association between MS and osteoporosis only in non-Asian subjects. In another meta-analysis, Xue et al. [13] also reported that MS may have a beneficial influence on BMD only in Caucasian populations. Genetic differences may explain the association between osteoporosis and MS be related to the source of the study population. The risk of osteoporosis has heritable factors, such as differences in bone geometry, size, and height [44, 45]. Third, we obtained a significant association between MS and osteoporosis only in the unadjusted model (univariate analysis). Although, in general, the adjusted models (multivariate analysis) were more accurate and deeper considered, we still accepted the unadjusted results to a certain extent. In the studies of the adjustment model, four datasets were adjusted for BMI, and one dataset was adjusted for serum total cholesterol. These adjustments distorted the clinical characteristics of MS, defined by obesity and dyslipidemia. In adjusting for these, the clinical sense of MS just disappeared or was at least essentially modified. However, this means that important confounding factors [46, 47] (such as BMI, physical activity, and dietary factors) are not controlled; this also is the reason that the finding should be interpreted with caution because the inverse associations of MS with osteoporosis risk could be attributed to the adoption of healthy lifestyles by subjects after being diagnosed MS.

Some limitations existed in our study. First, nonrandomized comparisons in observational studies may suffer from biases, which could impair the findings and thus weaken the strength of evidence. Second, most primary studies lacked data on important covariates, such as physical activities, vitamin *D* deficiency, and hormone therapy [48–50], which should be considered as a confounding factor when analyzing the association between MS and osteoporosis. Therefore, the risk of unmeasured confounding cannot be entirely ruled out. Third, although it would have been clinically meaningful to evaluate the effects of bone loss on different sites (total hip, femoral neck, and lumbar spine), we were unable to do so because of insufficient data. Fourth, in view of the heterogeneity, the random-effects model was used for meta-analyses, but in the comparison of normal BMD with decreased BMD, we included only four datasets in the analyses, so statistical power may be affected.

## 5. Conclusion

The findings of our meta-analyses suggested that individuals with MS demonstrate a lower risk of osteoporosis. Notably, subgroup analyses by gender showed that significant inverse associations were observed only in men but not in women. In addition, the association between osteoporosis and MS was likely to relate to the definition of MS, the source of the study population, and the adjustment of covariates. However, given the small number of studies mentioned above and the limitations of the study design, these findings must be interpreted carefully. The clinical significance of these findings remains uncertain and should be addressed in future well-designed prospective studies.

## Figures and Tables

**Table 1:** Characteristics of included studies.

Authors, year	Country (study period)	Study design	Sample size (male %)	Age (mean ± SD)	Metabolic syndrome diagnostic criteria	Outcome (diagnostic criteria)	BMD	
							Measurement	Site
Kim et al. 2013 [18]	Korean (2008–2010)	Cross-sectional study	3,207 (46.7)	52.0 ± 0.4	Harmonized criteria	Normal BMD vs. decreased BMD^a^ (WHO criteria)	DEXA	Femoral neck, lumbar spine, and total hip

Maghraoui et al. 2014 [19]	Morocco (2012–2013)	Cross-sectional study	270 (0.0)	61.0 ± 7.8	NCEPATP III	Nonosteoporosis^b^ vs. osteoporosis (WHO criteria)	DEXA	Femoral neck, lumbar spine, and total hip

Lee et al. 2015 [20]	Korea (2010–2011)	Cross-sectional study	3305 (46.5)	63.0 ± 8.4	NCEPATP III	Nonosteoporosis vs. osteoporosis (WHO criteria)	DEXA	Femoral neck and lumbar spine

Eckstein et al. 2016 [21]	Germany (2011–2014)	Cross-sectional study	1,402 (48.9)	68.1 ± 3.5	Harmonized criteria	Nonosteoporosis vs. osteoporosis (WHO criteria)	DEXA	Femoral neck, lumbar spine, and total hip

Abbasi et al. 2017 [22]	Iran (NA)	Cross-sectional study	143 (0.0)	56.8 ± 7.8	NCEPATP III	Nonosteoporosis vs. osteoporosis (WHO criteria)	DEXA	Femoral neck and lumbar spine

Heidari et al. 2017 [23]	Iran (2011–2012)	Cross-sectional study	553 (100.0)	70.7 ± 7.7	NCEPATP III	Nonosteoporosis vs. osteoporosis (WHO criteria)	DEXA	Femoral neck and lumbar spine

Chen et al. 2018 [15]	China (2011–2016)	Cross-sectional study	938 (0.0)	61.2 ± 13.8	CDS	Nonosteoporosis vs. osteoporosis (WHO criteria)	DEXA	Femoral neck, lumbar spine, and total hip

Loke et al. 2018 [13]	Taiwan (2014–2015)	Cross-sectional study	1162 (59.5)	59.9 ± 7.3	NCEPATP III	Normal BMD vs. decreased BMD (WHO criteria)	DEXA	Radius head, femoral neck, and total hip

Lin et al. 2018 [14]	Taiwan (NA)	Cross-sectional study	2007 (52.1)	58.9 ± NA	NCEPATP III	Nonosteoporosis vs. osteoporosis (WHO criteria)	DEXA	Femoral neck, lumbar spine, and total hip

*Note.* SD, standard deviance; BMD, bone mineral density; DEXA, dual-energy X-ray absorptiometry; NA, not available; CDS, Chinese Diabetes Society; NCEPATP III, the National Cholesterol Education Program Adult Treatment Panel III criteria. ^a^Decreased BMD includes both osteopenia and osteoporosis. ^b^Nonosteoporosis includes both normal BMD and osteopenia.

**Table 2:** Covariates adjusted for models of the associations between metabolic syndrome and osteoporosis.

Authors, year	Covariates
Kim et al. 2013 [18]	Men and premenopausal women adjusted for age, BMI, WBC count, alkaline phosphatase, smoking, alcohol intake, PHA, self-reported health status, daily calcium intake, chronic disease, rheumatoid arthritis, cancer, and parental osteoporosis; postmenopausal women further adjusted for years since menopause and postmenopausal hormone therapy

Maghraoui et al. 2014 [19]	Age, BMI, years since menopause, and number of pregnancies

Lee et al. 2015 [20]	Age, calcium intake, serum 25-OH vitamin *D* level, serum parathyroid hormone level, smoking status, alcohol consumption, PHA, hormone replacement therapy (in women), and muscle mass

Eckstein et al. 2016 [21]	None

Abbasi et al. 2017 [22]	None

Heidari et al. 2017 [23]	Age, BMI, muscle strength, PHA, educational level, history of fractures, abdominal obesity, smoking, and other biochemical parameters

Chen et al. 2018 [15]	Age, serum total cholesterol, alkaline phosphatase, and nonalcoholic fatty liver disease

Loke et al. 2018 [13]	None

Lin et al. 2018 [14]	Age, aspartate aminotransferase, creatinine, hemoglobin, and exercise status

*Note.* BMI, body mass index; WBC, white blood cell; PHA, physical activities.

**Table 3:** The methodological quality of the included studies.

Authors, year	1	2	3	4	5	6	7	8	9	10	11	Score	Quality level
Cross-sectional studies^a^													
Kim et al. 2013 [18]	1	1	1	1	0	0	1	1	1	1	1	9	High
Maghraoui et al. 2014 [19]	1	1	1	1	0	0	1	1	1	1	1	9	High
Lee et al. 2015 [20]	1	1	1	1	0	0	1	1	1	1	1	9	High
Eckstein et al. 2016 [21]	1	1	1	1	0	0	1	1	0	1	1	8	High
Abbasi et al. 2017 [22]	1	1	0	1	0	0	1	0	0	1	1	6	Moderate
Heidari et al. 2017 [23]	1	1	1	1	0	0	1	1	0	1	1	8	High
Chen et al. 2018 [15]	1	1	1	1	0	0	1	1	1	1	1	9	High
Loke et al. 2018 [13]	1	1	1	1	0	0	1	1	0	1	1	8	High
Lin et al. 2018 [14]	1	1	0	1	0	0	1	1	0	1	1	7	Moderate

^a^The Agency for Healthcare Research and Quality (AHRQ) was used to assess the study quality for cross-sectional studies.

**Table 4:** Subgroup analyses for comparing nonosteoporosis to osteoporosis with or without MS.

Group	No. of datasets	OR (95% CI)	*P*	*P* for heterogeneity	*I* ^2^ (%)
Definitions of MS					
NCEPATP III	7	0.67 (0.54, 0.84)	<0.001	0.253	23.1
CDS	1	3.16 (1.28, 5.21)	0.001	—	—
Harmonization criteria	2	0.54 (0.23, 1.30)	0.169	<0.001	82.1
Country					
Asian	7	0.86 (0.60, 1.23)	0.404	0.003	69.6
Non-Asian	3	0.46 (0.23, 0.89)	0.021	0.025	73.0
Quality of studies					
Moderate	3	0.80 (0.55, 1.15)	0.231	0.400	0
High	7	0.70 (0.45, 1.08)	0.108	<0.001	80.6
Confounding factors					
Adjusted	7	0.81 (0.54, 1.21)	0.303	<0.001	75.5
Unadjusted	3	0.54 (0.31, 0.95)	0.033	<0.061	64.2

*Note.* MS, metabolic syndrome; OR, odds ratio; CI, confidence interval; NCEPATP III, the National Cholesterol Education Program Adult Treatment Panel III criteria; CDS, Chinese Diabetes Society.

## Data Availability

The data used to support the findings of this study are included within the article.

## Abbreviations

AHRQ: Agency for Health Care Research and Quality
BMD: Bone mineral density
BMI: Body mass index
CDS: Chinese Diabetes Society
CI: Confidence interval
DEXA: Dual-energy X-ray absorptiometry
HDL-C: High-density lipoprotein cholesterol
MS: Metabolic syndrome
NCEPATP III: National Cholesterol Education Program's Adult Treatment Panel III
OR: Odds ratio
TG: Triglyceride
WHO: World Health Organization.
